# Visceral fat assessment in rectal adenocarcinoma: the role of computed tomography, sagittal abdominal diameter, and waist circumference

**DOI:** 10.1590/acb407925

**Published:** 2025-11-10

**Authors:** Daniela Vicinansa Monaco-Ferreira, Daniéla Oliveira Magro, Patrícia Prando Cardia, Claudia Luciana Fratta, Daniel Lahan Martins, Carlos Augusto Real Martinez, Cláudio Saddy Rodrigues Coy

**Affiliations:** 1 Universidade Estadual de Campinas – Faculdade de Ciências Médicas – Departamento de Cirurgia – Campinas (SP) – Brazil.; 2 Hospital Vera Cruz – Departamento de Radiologia – Campinas (SP) – Brazil.; 3 Universidade Estadual de Campinas – Faculdade de Ciências Médicas – Departamento de Radiologia – Campinas (SP) – Brazil.

**Keywords:** Colorectal Neoplasms, Sagittal Abdominal Diameter, Obesity, Abdominal, Tomography, Nutritional Assessment

## Abstract

**Purpose:**

To evaluate correlations between anthropometric tools for visceral fat assessment, including waist circumference (WC) and sagittal abdominal diameter (SAD), by computed tomography (CT)-derived visceral fat volume in patients with rectal adenocarcinoma.

**Methods:**

This comparative cross-sectional study included 138 participants: rectal adenocarcinoma patients (group 1, n = 69) and controls (group 2, n = 69). Assessed variables were weight, body mass index (BMI), bioelectrical impedance analysis (BIA), WC, SAD, and CT-derived visceral fat volume measured with Fat Tissue (Syngo.Via VB20, Siemens). CT analysis was restricted to group 1.

**Results:**

Median ages in groups 1 and 2 were 60 and 53 years, respectively (p < 0.0008). CT revealed sex-based differences in visceral fat: 20.91 cm^3^ in females and 31.29 cm^3^ in males (p = 0.0043). WC and SAD demonstrated statistically significant correlations with CT-derived visceral fat in group 1 (p < 0.0001).

**Conclusion:**

WC and SAD correlated with CT-derived visceral fat in rectal adenocarcinoma patients. These exploratory findings require validation by larger studies with multivariable analyses to establish predictive value and clinical applicability.

## Introduction

In developing countries, there has been a decline in infection-related cancers and a parallel rise in malignancies associated with socioeconomic changes and urbanization^1^. In the United States of America, recent data indicated that colorectal cancer (CRC) is the third leading cause of cancer-related death^2^. Factors such as food intake and sedentary behavior, both considered modifiable lifestyle factors, play a significant role in the development and progression of CRC^3^^–^ 8.

The association between obesity and CRC has prompted growing interest in identifying accurate methods for assessing body composition, particularly visceral fat and muscle mass^9^^–^ 18. These parameters can influence outcomes related to surgery, chemotherapy, quality of life, comorbidities, and overall survival^19^^–^ 25.

Several tools are available to assess visceral adiposity, including waist circumference (WC), bioelectrical impedance analysis (BIA), dual-energy X-ray absorptiometry, magnetic resonance imaging (MRI), and computed tomography (CT)^11^^,^ 18 ^,^ 19 ^,^ 26 ^–^ 29. Each method has its advantages and limitations when applied in clinical settings. Sagittal abdominal diameter (SAD), a relatively recent anthropometric measure, has been proposed as a non-invasive predictor of visceral fat mass. However, most research on SAD has focused on populations with insulin resistance, cardiometabolic syndrome, or diabetes mellitus^30^^–^ 32.

In oncology, CT is considered the gold standard for assessing body composition, enabling accurate evaluation of sarcopenia and visceral obesity, as well as monitoring changes in muscle mass in CRC patients^18^^,^ 19 ^,^ 26 ^,^ 27 ^,^ 33 ^–^ 35. Nevertheless, access to CT and other advanced technologies is limited in many clinical settings.

Therefore, identifying alternative and accessible methods to estimate visceral fat may help improve risk stratification, treatment response prediction, and perioperative planning in patients with rectal adenocarcinoma. This study aimed to correlate anthropometric measures (WC and SAD) and bioelectrical impedance analysis with CT-based visceral fat volume in patients with rectal adenocarcinoma.

## Methods

This comparative and cross-sectional study included patients diagnosed with rectal adenocarcinoma (group 1) and their non-consanguineous household companions (group 2). The study was conducted at the Colorectal Cancer Outpatient Clinic of the Universidade Estadual de Campinas (Campinas, SP, Brazil) between August 2017 and December 2019. Participants were recruited using convenience sampling; all patients accompanied by a non-consanguineous companion on the day of consultation were invited to participate.

Inclusion criteria for group 1 comprised individuals diagnosed with rectal adenocarcinoma, regardless of whether they had received neoadjuvant treatment, provided they were accompanied by a non-consanguineous companion and consented to participate. Group 2 included individuals with no history of rectal adenocarcinoma who lived in the same household as the group 1 participants. This group was included to enable comparison of body composition and lifestyle habits between individuals sharing the same environment.

Exclusion criteria included a history of intestinal resection, synchronous tumors, metastases, inflammatory bowel disease, familial adenomatous polyposis, Lynch syndrome, cognitive impairment, age under 18 years old, wheelchair use, or lack of a companion at the time of consultation.

### Variables and definitions

Clinical variables included age, sex, race, educational level, smoking status, alcohol consumption (defined as one dose per day), comorbidities (diabetes mellitus, arterial hypertension, and dyslipidemia), and physical activity (defined as ≥ 75 minutes per week). Tumor staging followed the 8th edition of the American Joint Committee on Cancer guidelines^36^.

Anthropometric measurements included weight, height, body mass index (BMI, kg/m^2^), WC (cm), BIA (fat mass in kg and % body fat), SAD (cm), and visceral fat volume assessed via CT (cm^3^).

BIA was performed by a tetrapolar bioimpedance analyzer (Biodynamic Body Composition Analyzer, model 310, Biodynamics Corporation, Seattle, WA, United States of America). Measurements were taken with participants in the supine position on a non-conductive surface, with electrodes placed on the right side upper and lower limbs.

SAD was measured by a Holtain-Kahn abdominal caliper (Holtain, Wales, United Kingdom) with a movable rod and 0.1-cm scale. Participants were positioned supine with knees flexed, and the caliper was applied in the sagittal plane. The vertical alignment was verified by the air bubble indicator in the upper stem^30^^,^ 32.

BMI was calculated and categorized according to the World Health Organization guidelines^37^. WC was classified based on BMI and sex^38^ and measured with a flexible and inelastic measuring tape. Body fat percentage was assessed by BIA and interpreted according to the Lohman classification by sex and age^39^. All nutritional assessments were performed by a single trained nutritionist to ensure consistency.

### Assessment of visceral fat

SAD and CT were used to evaluate visceral fat. SAD measurements were taken at two anatomical points:

- The site of the greatest abdominal diameter^40^;
- The narrowest point between the thorax and hips^41^.

Due to the lack of reference standards in the CRC population, sex-specific cut-off points were derived from the study sample median: 19.3 cm for women and 20.5 cm for men^42^.

CT-based evaluation of visceral fat was performed only in group 1, as part of routine oncologic staging. Images were retrieved from existing staging scans using 64-channel (Aquilion, Toshiba/Canon Medical, Ōtawara, Japan) or 16-channel (BrightSpeed, GE Healthcare, Chicago, United States of America) scanners. A single axial slice at the umbilical level, corresponding to the third lumbar vertebra (L3), was used for analysis, as this region is considered representative of total visceral fat volume^18^^–^ 20.

**Figure 1:**
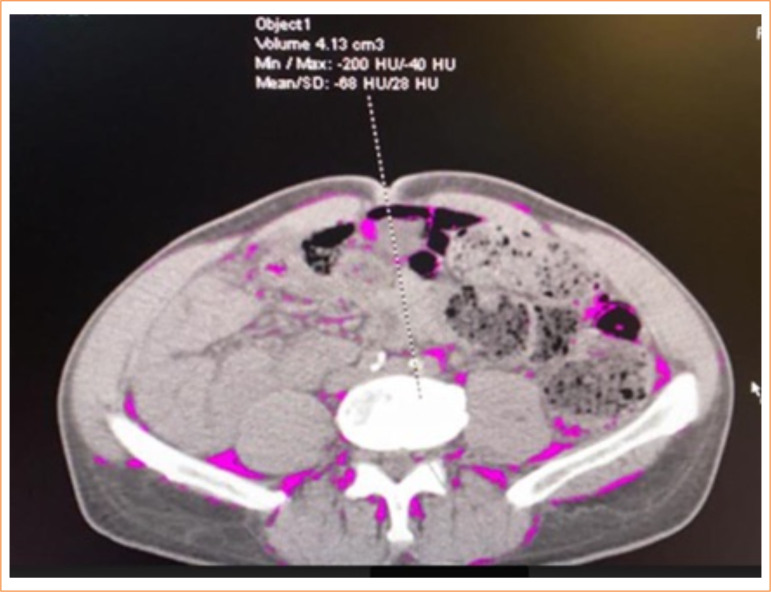
Representative computed tomography scan image at the L3 vertebral level in a patient with rectal adenocarcinoma, showing a low volume of visceral fat, with the pink area indicating the amount of visceral fat in cm^3^.

Source: Elaborated by the authors.

**Figure 2:**
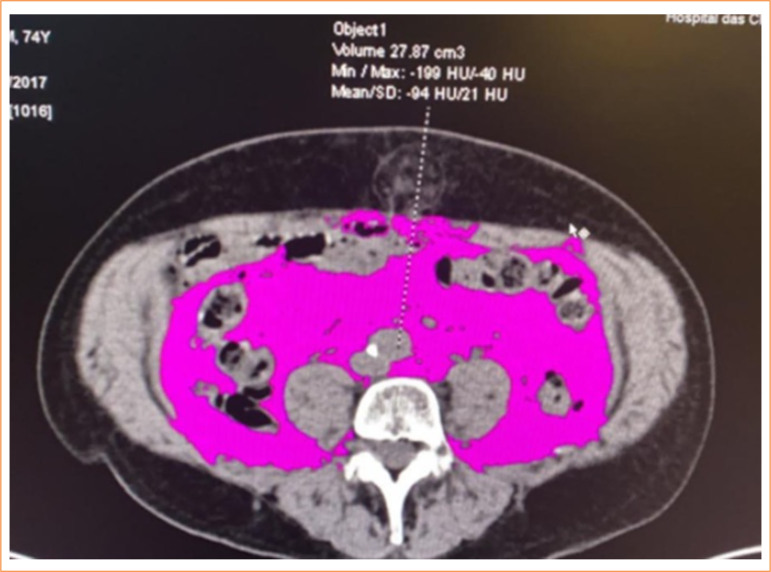
Representative computed tomography scan image at the L3 vertebral level in a patient with rectal adenocarcinoma, showing a high volume of visceral fat, with the pink area indicating the amount of visceral fat in cm^3^.

Source: Elaborated by the authors.

Visceral fat volume was quantified using the Fat Tissue software (Syngo.Via VB20 platform, Siemens, Erlangen, Germany)^43^. The visceral fat area was manually outlined along the inner borders of the rectus abdominis, internal oblique, and quadratus lumborum muscles, including retroperitoneal, mesenteric, and omental fat, while excluding the vertebral body and paravertebral muscles. A histogram-based algorithm using radiodensity thresholds between -40 and -150 Hounsfield units was applied^19^^,^ 44. The results were expressed in cubic centimeters (cm^3^) (Figs. 1 and 2).

Measurements were independently conducted in duplicate by two experienced radiologists blinded to group assignment and anthropometric data. Discrepancies were resolved by consensus. Each radiologist verified their own segmentations to ensure intra-rater consistency and reproducibility.

This study was approved by the Research Ethics Committee (CAAE: 6034316.20000-5404; Opinion No. 1967.445), and all participants provided written informed consent.

### Data analysis

Statistical analyses were performed by SAS software version 9.4 (SAS Institute, Cary, NC, United States of America) for Windows 2002–2012^45^. In addition to median analysis of visceral fat volume, the 10th, 25th, 75th, and 90th percentiles were also calculated^46^. Proportions were compared by the χ^2^ test or Fisher’s exact test, as appropriate. Continuous variables were compared between groups using the Mann–Whitney U test^46^. Associations between continuous variables were assessed by Spearman’s rank correlation coefficient^46^.

Inter-rater agreement between radiologists was calculated by the intraclass correlation coefficient. *P* < 0.05 was considered statistically significant.

## Results

### Patients’ characteristics

A total of 138 participants were included and divided into two groups: those with CRC (group 1, n = 69) and those without a cancer diagnosis (group 2, n = 69).

The main clinical and demographic characteristics of the patients are summarized in Table 1. Significant differences were observed between the groups in terms of age (*p* = 0.0008) and sex (*p* = 0.0012). In group 1, 63.8% were male. Regarding smoking, 44.9% of participants with rectal cancer were former smokers for less than six months (*p* = 0.0436). There was a predominance of T3 tumors (39.13%) with positive lymph node involvement. Among the participants in group 1, 72.5% had not undergone neoadjuvant therapy at the time of evaluation. Approximately 70% of individuals in both groups did not engage in physical activities, and alcohol consumption was more frequent in group 2 (52.2%).

**Table 1:** Characteristics of patients with rectal cancer and controls without the disease.

Variables	Group 1 (CRC patients) (n = 69)	Group 2 (without the illness) (n = 69)	*p*-value
**Age in years, median** (minimum/maximum)	60.00 (30.00–84.00)	53.00 (24.00–77.00)	0.0008^1^
**Sex, n (%)**			**0.0012** ^**2**^
Male	44 (63.8)	25 (36.2)	
Female	25 (36.2)	44 (63.8)	
**Race, n (%)**			0.1524^3^
White	56 (81.2)	58 (84.1)	
Black	5 (7.2)	9 (13.0)	
Mixed	6 (8.7)	1 (1.4)	
Asian	2 (2.9)	1 (1.4)	
**Level of education**			0.4059^3^
Middle school	32 (46.4)	29 (42.0)	
High school	20 (29.0)	26 (37.7)	
College	11 (15.9)	12 (17.4)	
Illiterate	6 (8.7)	2 (2.9)	
**Smoking**			**0.0436** ^**2**^
Non-smoker	32 (46.4)	44 (63.8)	
Ex-smoker	31 (44.9)	17 (24.6)	
Smoker	6 (8.7)	8 (11.6)	
**Alcoholism**			0.1721^2^
No	41 (59.4)	33 (47.8)	
Yes	28 (40.6)	36 (52.2)	
Comorbidities*			0.8627^2^
No	28 (40.6)	29 (42.0)	
Yes	41 (59.4)	40 (58.0)	
			0.0569^3^
HTN	17 (24.6)	20 (29.0)	
DM	0 (0.0)	4 (5.8)	
Dyslipidemia	4 (5.8)	3 (4.3)	
HTN/DM/associated	14 (20.3)	4 (5.8)	
Others	6 (8.7)	9 (13.0)	
**Physical activity**			0. 6928
No	53 (76.8)	51 (73.9)	
Yes	16 (23.2)	18 (26.1)	
**Cancer stage**			
0	1 (1.44)		
I	13 (18.84)		
II A	5 (7.25)		
III A	4 (5.8)		
III B	27 (39.13)		
III C	19 (27.54)		
**Treatment**			
None	50 (72.5)		
ChT and RT	15 (21.7)		
ChT	3 (4.3)		
RT	1 (1.4)		

1 Mann-Whitney test1;

2 χ^2^ test;

3 Fisher’s exact test;

CRC: colorectal cancer; ChT: chemotherapy; RT: radiotherapy; HTN: Hypertension; DM: diabetes mellitus. Source: Elaborated by the authors.

### Assessment of body composition

Table 2 shows that there was no significant difference in age between females with and without CRC (*p* = 0.0782). However, males with CRC were significantly older than those in the control group (*p* = 0.0170). The percentage of weight loss was higher in both males (*p* < 0.001) and females (*p* < 0.001) with CRC.

**Table 2:** Anthropometric measurements and body composition parameters by bioimpedance, sagittal abdominal diameter among the groups, and visceral fat volume for group 1.

Variables	Groups with and without the disease	Median (minimum/maximum)	*p*-value
**Age**	Group 1 Female N = 25	56.00 (30.00–83.00)	0.0782^1^
	Group 2 Female N = 44	51.00 (24.00–77.00)	
	Group 1 Male N = 44	60.50 (39.00–84.00)	**0.0170** ^1^
	Group 2 Male N = 25	54.00 (29.00–71.00)	
**Anthropometry**			
**%WL**	Group 1 Female N = 25	6.25 (0.00–26.67)	**< 0.0001** ^1^
	Group 2 Female N = 44	0.00 (0.00–18.75)	
	Group 1 Male N = 44	6.74 (0.00–24.00)	**< 0.0001** ^1^
	Group 2 Male N = 25	0.00 (0.00–18.06)	
**BMI**	Group 1 Female N = 25	25.15 (17.90–47.22)	0.3396^1^
	Group 2 Female N = 44	28.74 (19.40–37.20)	
	Group 1 Male N = 44	25.98 (19.93–39.56)	0.1216^1^
	Group 2 Male N = 25	28.73 (16.90–37.40)	
**WC**	Group 1 Female N = 25	95.00 (65.00–127.00)	0.4025^1^
	Group 2 Female N = 44	98.00 (67.00–124.00)	
	Group 1 Male N = 44	99.00 (78.00–136.00)	0.3488^1^
	Group 2 Male N = 25	101.00(70.00–134.00)	
**Bioimpedance**			
**Fat mass (kg)**	Group 1 Female N = 25	23.10 (8.60–61.60)	0.2692^1^
	Group 2 Female N = 44	26.20 (9.40–48.30)	
	Group 1 Male N = 44	19.90 (7.20–58.00)	0.1941^1^
	Group 2 Male N = 25	23.90 (6.20–66.40)	
**% of fat**	Group 1 Female N = 25	34.30 (17.50–48.50)	0.6352^1^
	Grupo 2 Female N = 44	35.90 (20.40–45.70)	
	Group 1 Male N = 44	27.00 (9.60–39.90)	0.5743^1^
	Group 2 Male N = 25	27.80 (12.40–36.40)	
**Sagittal abdominal diameter**			
**SAD 1**	Group 1 Female N = 25	22.50 (16.00–28.50)	0.8367^1^
	Group 2 Female N = 44	22.65 (18.00–29.00)	
	Group 1 Male N = 44	25.00 (18.50–32.00)	0.6213^1^
	Group 2 Male N = 25	24.50 (18.50–32.80)	
**SAD 2**	Group 1 Female N = 25	22.00 (13.00–30.50)	0.8660^1^
	Group 2 Female N = 44	21.00 (13.00–30.00)	
	Group 1 Male N = 44	21.75 (15.50–31.80)	0.2003^1^
	Group 2 Male N = 25	23.00 (16.50–34.00)	
**Fat volume**	**Group 1 Female N = 25**		**0.0043** ^1^
	P 10	9.12	
	P 25	12.28	
	P 50	20.91	
	P 75	39.01	
	P 90	46.34	
	**Group 1 Male N = 44**		**0.0043** ^1^
	P 10	18.12	
	P 25	22.64	
	P 50	31.29	
	P 75	58.30	
	P 90	67.88	

1 Mann-Whitney test; group 1: colorectal cancer patients; group 2: without the illness;

%WL: percentage of weight loss; BMI: body mass index, in kg/m^2^; WC: waist circumference, in cm; SAD 1: largest abdominal diameter; SAD 2: smallest waist between chest and hips. Source: Elaborated by the authors.

No group differences were observed in BMI, WC, fat mass, body fat percentage, or SAD, regardless of sex.

Visceral fat volume in the CRC group varied across percentiles. At the 50th percentile, males had a higher visceral fat volume than females (31.29 *versus* 20.91 cm^3^; *p* = 0.0043). Both groups were classified as overweight, with WC and body fat percentage above recommended thresholds.

### Assessment of visceral fat according to computed tomography and its relationship with anthropometric parameters

**Figure 3:**
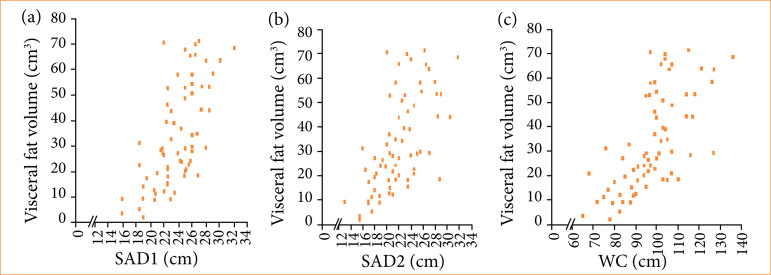
Colorectal cancer patients group: **(a)** Spearman’s linear correlation (0.66) for measurements of visceral fat volume by tomography and the largest sagittal abdominal diameter (SAD 1) (*p* < 0.001); **(b)** Spearman’s linear correlation (0.66) for measurements of visceral fat volume by tomography and SAD referring to the smallest waist between chest and hips (SAD 2) (*p* < 0.001); **(c)** Spearman’s linear correlation (0.70) for measurements of visceral fat volume by tomography and waist circumference (*p* < 0.001).

Source: Elaborated by the authors.

Volumetric measurements of visceral adipose tissue were correlated with anthropometric parameters in participants with CRC (Fig. 3). A moderate correlation was observed between visceral fat volume and SAD, while WC showed a strong correlation with CT-derived visceral fat volume.

**Figure 4:**
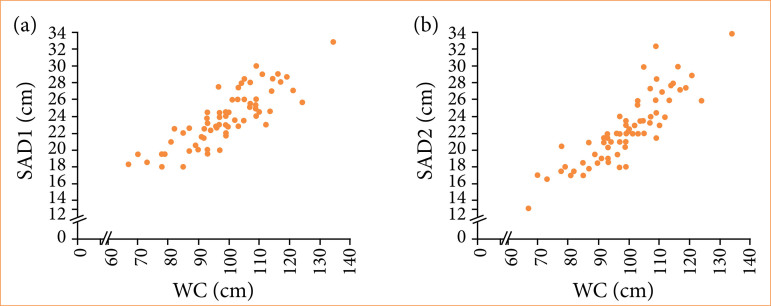
Control group: **(a)** Spearman’s linear correlation (0.84) for measurements of the largest sagittal abdominal diameter (SAD 1) and waist circumference (*p* < 0.001); **(b)** Spearman’s linear correlation (0.89) for measurements of sagittal abdominal diameter at the smallest waist between the chest and hips (SAD 2) and waist circumference (*p* < 0.0001).

Source: Elaborated by the authors.

In the control group, a strong correlation was also observed between SAD and WC (Fig. 4).

The inter-rater agreement for CT-based visceral fat measurements performed by the two radiologists was excellent, with an intraclass correlation coefficient = 0.982, indicating high measurement reproducibility.

## Discussion

This is the first study to apply SAD to assess visceral adiposity in patients with CRC, using theoretical and empirical references from cardiometabolic populations^30^^–^ 32. These thresholds, originally validated in individuals with insulin resistance, cardiometabolic risk, and type-2 diabetes, were adopted based on shared pathophysiological mechanisms such as visceral adiposity, chronic inflammation, and metabolic dysregulation. Although SAD has been validated in cardiometabolic cohorts, its use in oncologic populations, particularly in older adults and patients receiving cancer treatment, remains scarcely investigated. Furthermore, the demographic imbalance between groups (predominantly older males in the cancer group and younger females in the control group) constitutes a structural confounding bias that should be carefully considered when interpreting intergroup comparisons. This gap underscores the relevance of our study, which provides exploratory evidence on the applicability of SAD in rectal adenocarcinoma.

Our results demonstrated that both SAD measurements (the largest abdominal diameter and the smallest waist between the thorax and hips) were positively correlated with CT-derived visceral fat in patients with CRC. Among the anthropometric tools evaluated, WC showed the strongest correlation with CT, whereas SAD presented a moderate association.

Although SAD is a relatively recent anthropometric indicator, its use has primarily been limited to populations at cardiometabolic risk. Nevertheless, its application in CRC is plausible due to shared pathophysiological mechanisms with metabolic syndrome, including visceral adiposity, insulin resistance, and chronic inflammation. In this context, SAD may serve as a viable surrogate marker for estimating visceral fat in individuals with CRC. This study provides exploratory reference values for SAD in this population and emphasizes the need for further validation in larger and more diverse oncologic cohorts.

The BMI profile of patients with CRC prior to diagnosis reflected an overweight status, followed by weight loss exceeding 5%. According to the European Society for Clinical Nutrition and Metabolism guidelines^47^, a weight loss greater than 5% is associated with nutritional risk, reduced quality of life, and adverse clinical outcomes, even in individuals with obesity. These findings underscore the need for accessible tools to screen for nutritional risk early in oncology care, particularly in settings in which CT is unavailable. Anthropometric indicators such as WC and SAD could complement nutritional assessment when properly validated.

Barrett et al.^11^ emphasized the importance of considering weight loss history, nutritional indices, and body composition via CT in oncology. Similarly, Martin et al.^48^ demonstrated that a greater association between weight loss percentage and BMI is linked to reduced survival, regardless of cancer stage or site. Purcell et al.^49^ further noted that weight loss negatively impacts survival, while weight gain does not confer a protective effect. Several studies have shown that visceral obesity is associated with an increased risk of CRC, adverse treatment outcomes, and higher complication rates^18^^,^ 25 ^,^ 27 ^,^ 33 ^,^ 50. In patients with CRC, visceral adipose tissue has been linked to higher infection rates, postoperative complications, and increased hospital readmissions^8^^,^ 10 ^,^ 26. Various methods have been proposed to assess visceral obesity. It remains crucial to establish a simple and accessible technique that correlates with visceral fat and can be used clinically to anticipate complications and guide nutritional management in CRC care.

The relevance of CT in evaluating visceral fat and lean mass in patients with CRC is well established^10^^,^ 14 ^,^ 17 ^–^ 20. However, CT is not universally accessible or cost-effective in all clinical settings. This exploratory study identified strong correlations between CT-derived visceral fat volume and accessible anthropometric measures such as WC and SAD in patients with CRC. These findings support the potential utility of these indicators in clinical practice, particularly in settings in which CT is not available. It is important to note that all patients evaluated were at the beginning of treatment and did not present conditions such as ascites, which might compromise anthropometric measurements.

Although WC has been widely validated as a surrogate for visceral adiposity and cardiometabolic risk, the present study identified a strong correlation between WC and CT-derived visceral fat volume in patients with CRC. According to the most recent consensus on visceral obesity^38^, WC is considered a key indicator, and its predictive value is further enhanced when adjusted for BMI and sex.

Maurovich-Horvat et al.^44^ compared anthropometric indicators, including WC, BMI, and SAD, with CT-derived measures of subcutaneous and visceral abdominal adipose tissue volumes in 100 healthy Caucasian individuals (51% male; aged 37–83 years old). The authors found strong volumetric correlations between anthropometric and CT measures (*p* < 0.0001). A similar finding was observed in the present study, although the correlation between SAD and CT-based visceral fat volume was lower in patients with CRC.

Kvist et al.^51^ demonstrated that CT-based assessments reliably measure both total and visceral adipose tissue volume in adults, and that slices taken at L4–L5 and L3–L4 show comparable correlations in both sexes (*p* < 0.001). However, standardized cut-off points for assessing visceral fat specifically in CRC patients have yet to be established^34^^,^ 52. In our study, CT images were obtained at the level of the umbilicus, corresponding to the third lumbar vertebra (L3), which has been shown to correlate strongly with total visceral fat volume^8^^,^ 10 ^,^ 11 ^–^ 13 ^,^ 19 ^–^ 21 ^,^ 23 ^,^ 24 ^,^ 26 ^,^ 27 ^,^ 33 ^,^ 35. Results were stratified by percentiles and sex, and higher visceral fat volume was consistently observed in males across all percentiles.

The application of sophisticated techniques to assess visceral obesity in oncology is a growing trend and has proven valuable for evaluating therapeutic response, prognosis, and overall survival^53^. Nonetheless, practical tools that can be implemented in routine clinical practice are necessary, especially in resource-limited settings in which CT may not be available.

CT remains the gold standard for body composition analysis and has been increasingly utilized for nutritional assessment in CRC patients^10^^,^ 14 ^,^ 17 ^–^ 20. Similar to our findings, Nattenmueller et al.^54^ reported higher visceral fat levels in males compared to females and demonstrated a positive association between waist-to-height ratio and CT-based fat volume. While sex-based differences in body composition are well documented, our study reinforces this pattern in a CRC population using a gold standard method and highlights the feasibility of incorporating accessible anthropometric measures in clinical settings.

Previous research has employed anthropometric markers and CT-derived fat volume to predict postoperative outcomes following CRC surgery^55^^,^ 56. These studies have shown that visceral fat, particularly when assessed through waist-to-hip and waist-to-height ratios, is predictive of intraoperative complications, medical events, and reinterventions when compared to CT^55^. In this context, the present study underscores the relevance of WC and SAD as low-cost and accessible anthropometric tools that correlate with CT-derived visceral fat volume in patients with rectal adenocarcinoma. Given their simplicity, these indicators may support future research in preoperative risk stratification and treatment planning. However, as this was an exploratory study based on correlation analyses, we did not assess the predictive accuracy or clinical applicability of these measures. Therefore, although WC and SAD may contribute to future prognostic models or decision-making tools, further validation is required through receiver operating characteristic (ROC) curves and multivariable regression analysis.

Another strength of this study was the use of WC adjusted for BMI and sex to predict visceral fat in patients with CRC, as recommended by the visceral adiposity consensus^38^.

According to the latest consensus on visceral obesity^38^, the predictive value of WC for morbidity and mortality is strengthened when adjusted for BMI, making it a reliable indicator of increased visceral adiposity in adults.

The presence of differences in age and sex between groups constitutes a source of structural confounding, as both factors independently influence visceral fat distribution irrespective of BMI. Given the small sample size and the non-parametric distribution of the main variables, multivariable adjustment (*e.g.*, regression analysis) was not feasible. Consequently, intergroup comparisons should be regarded as exploratory, and the main conclusions of this study are restricted to intragroup analyses in the cancer cohort. The use of convenience sampling, although practical in the outpatient setting, may have introduced selection bias and residual confounding due to shared environmental factors such as diet and physical activity, particularly since control participants were household members. Moreover, CT imaging was performed exclusively in the CRC group (group 1), as it would have been ethically inappropriate in healthy controls. The prospective design also precluded the inclusion of controls with pre-existing CT scans obtained for non-oncological reasons, a strategy commonly feasible in retrospective observational studies, which further limited external validity and intergroup comparability. Non-parametric statistical tests were employed, which prevented adjustment for key confounding variables such as age, sex, BMI, or neoadjuvant treatment.

Although this study demonstrated correlations between WC, SAD, and CT-derived visceral fat volume in patients with rectal adenocarcinoma, it was not designed to develop or validate predictive models. ROC curve analyses and multivariable regression were not performed to establish diagnostic thresholds or assess sensitivity and specificity. Therefore, the present findings should be considered exploratory and hypothesis-generating. Future prospective studies with larger and more diverse cohorts, incorporating multivariable regression and ROC curve analyses, are required to validate these associations, establish diagnostic thresholds, and determine the independent predictive value and potential clinical applicability of anthropometric indicators such as WC and SAD in oncologic practice.

## Conclusion

WC and SAD were correlated with CT-derived visceral fat volume in patients with rectal adenocarcinoma. Given the exploratory design and the absence of multivariable adjustment for potential confounders, these findings should be considered preliminary and interpreted with caution. Future prospective studies with larger and more diverse cohorts, incorporating multivariable regression and ROC curve analyses, are needed to confirm these associations, define diagnostic thresholds, and determine the independent predictive value and potential clinical utility of WC and SAD in oncologic practice.

The study was conducted under the Declaration of Helsinki and approved by the Research Ethics Committee of the Faculty of Medical Sciences of the Universidade Estadual de Campinas, opinion number 1,967,445, of March 16, 2017. Resolution CNS 196/96 considered, described, and established rules and regulations for research involving human beings.

## Data Availability

The original data presented in this study are publicly available in the open-access repository in which Daniela Vicinansa Monaco Ferreira’s doctoral thesis, entitled “Nutritional and Body Composition Assessment of Rectal Adenocarcinoma Carriers,” is archived. Handle: T/UNICAMP M741a.
